# Shear strength characteristics of a sand clay liner

**DOI:** 10.1038/s41598-020-75188-1

**Published:** 2020-10-26

**Authors:** Muawia Dafalla, Abdullah Shaker, Tamer Elkady, Abdullah Almajed, Mosleh Al-Shamrani

**Affiliations:** 1 Bughshan Research Chair in Expansive Soils, King Saud University, Civil Engineering, Riyadh, 11421 Saudi Arabia; 2 Civil Engineering, King Saud University, Riyadh, 11421 Saudi Arabia

**Keywords:** Environmental sciences, Natural hazards, Solid Earth sciences, Engineering, Materials science

## Abstract

This study investigated shear strength behaviour of compacted sand–clay mixtures used as liners, with 10%, 20%, and 30% clay contents. A natural high-plasticity and highly expansive clay found in the eastern province of Saudi Arabia was used. A series of consolidated undrained triaxial tests and pore water pressure measurements of saturated samples with various clay contents and confining pressures was conducted using a computer-controlled Bishop and Wesley triaxial cell. The unit was equipped with pressure volume controllers and a pressure transducer for measuring sample volume changes and excess pore water pressure. The experimental test results indicate that clay content and confining pressure significantly affect stress strain response curves, pore water pressure generation curves, and steady-state shear strength. Sand–clay mixtures with clay content less than 10% showed a tendency toward contractive behaviour. The failure line slope increased in accordance with clay content increase. The deviator stress versus axial strain of saturated sand–clay mixtures indicated a hyperbolic trend. The stress ratio versus axial strain representation was more informative for the shear strength behaviour assessment. Clay content did not significantly affect critical-state friction angle. Scanning electron microscope images of the sand-clay mixtures with different clay contents are presented.

## Introduction

In engineering, high-plasticity sand–clay mixtures are typically used as hydraulic barriers in waste containment systems and irrigation canals and as impermeable cores in earth dams. In these mixtures, the coarse grain fraction controls the soil shear strength behaviour, and hydraulic conductivity is expected to be governed by the fine-grained proportion. The main stream of research conducted to investigate the shear strength behaviour of sand–clay mixtures has focused on the influence of the clay or sand content on the shear strength magnitude and parameters of mixtures^1–7^. Studying the shear strength of the material used in liners is greatly important for the choice of the liner material. This will help in designing the top layers subjected to possible traffic or landfills involving large overburden pressure or steep slopes. These studies have shown three shear strength behaviour of sand–clay mixtures as a function of the clay content: non-cohesive behaviour where cohesion is considered negligible, and the friction angle is above 30°; transitional behaviour, and cohesive behaviour where cohesion makes an appreciable contribution to the shear strength. Many studies have described the impact of different clay fractions on the shear strength behaviour. The stress–strain behaviour of clay–sand mixtures is significantly influenced by gradations and proportions of mix constituents^8^. Simpson and Evans ^9^ discussed the behavioural thresholds in sand and kaolinite clay mixtures. The nonplastic silt content in sand–silt mixtures can have an influence that is different from clay as presented in^10,11^ used advanced isotropic testing and concluded that the net mean stress affects the stiffness, undrained shear strength, and deformation of the 30% bentonite content of a selected bentonite sand mixture. Reference^12^ stated that sand clay mixtures with a clay content of less than 10% follow the compression curve of sand at mean effective stresses greater than 30 MPa. Moreover, mixtures of more than 20% approach the compression curve of pure clay. No detailed studies in technical literature have yet been presented with regard to the factors controlling the clay fraction behaviour.

Only a few studies have investigated the pre-failure and failure characteristics of sand–clay mixtures during shear. Reference^13^ reported that the clay content in clayey sand had a notable effect on the compression and extension triaxial test behaviour. The dilatant behaviour of the clayey sand decreases with the increase of fines. Reference^5^ reported that the shear strength properties and stress–strain characteristics of sand–kaolin mixtures exhibit a significant change at 20% kaolin content. Meanwhile, Ref^14^ experimentally studied the behaviour of sand–clay mixtures under compression and extension for variable load paths. The experiment results revealed that the anisotropy in mechanical behaviour was greater in compression than in the extension tests when considering the shear stress parameters^14^. Chen and Mechan^15^ performed a series of unconsolidated undrained triaxial tests to evaluate the effects of the bentonite content, compaction moisture content, compaction energy, and confining pressure on the shear strength and stress–strain behaviour of the compacted sand and bentonite mixtures. Their experimental results showed that the undrained shear strength decreases with the increase in the compaction water content, but increases with the increase in the bentonite content, compaction effort, and confining pressure. In the same study, the compacted dry-of-optimum samples exhibited a stiffer and more brittle behaviour than the compacted wet-of-optimum samples.

The present study aims to investigate the pre-failure and failure characteristics of saturated remolded sand clay mixtures with different clay contents (i.e., 0, 10%, 20%, and 30%) under undrained monotonic compression loading. The high-plasticity clay used herein was obtained from the eastern province of the Kingdom of Saudi Arabia. Several previous studies on the characteristics of this clay revealed that it was highly expansive^16–19^. The influence of the clay contents (i.e., 0, 10%, 20%, and 30%) and the effective confining pressure ranging from 25 to 100 kPa on the pore pressure generation, stress paths, and steady-state shear strength is discussed accordingly.

## Materials and testing methods

### Materials

The clay used in this study was an expansive, high-plasticity clay found in Al-Qatif City, which is located in the coastal areas of the eastern province of the Kingdom of Saudi Arabia (26° 56′ 0″ N, 50° 1′ 0″ E). Disturbed samples of greenish brown clay were obtained from open pits excavated to 2.5–3.0 m depth below the ground surface. Extensive laboratory tests were performed to characterize the soil swelling characteristics of the Al-Qatif soils (Table 1). The sand used herein was uniform sand with a grain size ranging from 0.15 to 0.6 mm and classified as poorly graded sand (SP) as per the unified soil classification system.

**Table 1:** Geotechnical characteristics of the Al-Qatif clay.

Property	Value
**Index parameters**	
Specific gravity, G_s_	2.75
Liquid limit, LL (%)	140
Plastic limit, PL (%)	45
Plasticity index, PI (%)	95
USCS	CH^a^
**Swelling characteristics**	
Swelling pressure (kN/m^2^)	550–600
Swelling potential (%)	16–18

^a^Clay with high plasticity.

### Mixture and sample preparation

The Al-Qatif clay obtained in the field was air-dried, pulverized, and sieved using sieve No. 40. The targeted proportions of the oven-dried sand and clay were mixed by hand under dry conditions to obtain a homogenous blend. Water was then added, and the mixture was thoroughly mixed to achieve the required moisture content. Subsequently, the samples were stored in plastic bags placed in an air-tight container, for at least 24 h for curing. The specimens were then prepared by static compaction in stainless split mods to final dimensions of 50 mm diameter and 100 mm height. The target dry unit weight and the moisture content used to prepare the sand–clay mixtures with different clay contents corresponded to the maximum dry unit weight and the optimum moisture content deduced from the Standard Proctor compaction curves according to ASTM D 698^20^. Table 2 summarizes the initial dry unit weight and the water content of the tested specimens.

**Table 2:** Initial dry unit weight and moisture content for the sand–attapulgite clay mixtures.

Clay content (%)	γ_dmax_ (kN/m^3^)	*w* (%)
0	16.22	10.5
10	17.85	11
20	18.15	13
30	18.03	13.5

### Testing methodology

**Figure 1:**
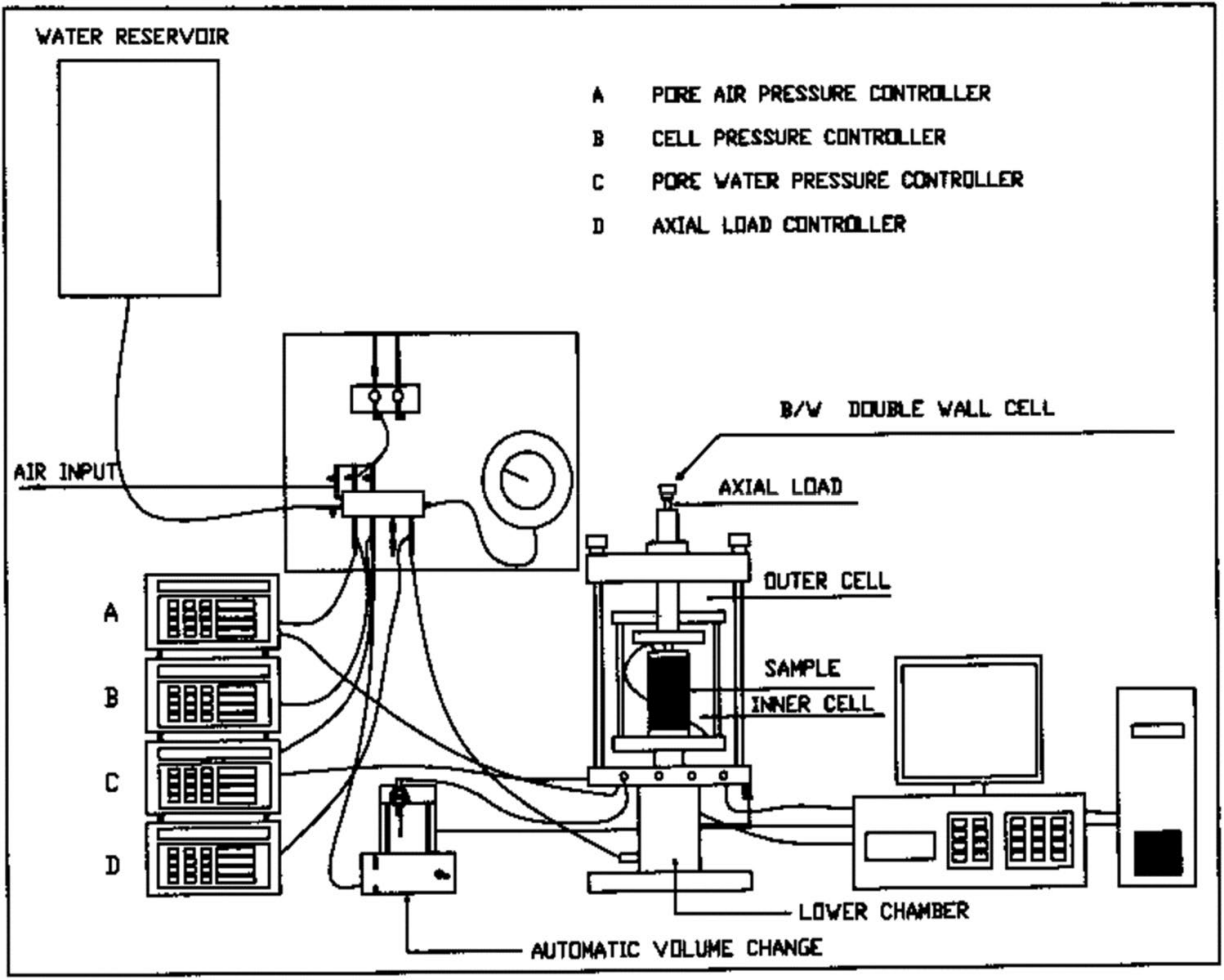
Schematic layout of the testing system.

A series of isotopically consolidated undrained compression tests was performed to evaluate the undrained shear strength of the sand–clay mixtures. The tests were performed using a computer-controlled Bishop and Wesley triaxial cell. Back pressure and cell pressures were controlled using a pressure volume controller. The pore pressures generated in the specimen samples during shearing were recorded using a pressure transducer. The axial load was measured using a submersible load cell. Meanwhile, the axial deformation was measured using an external linear variable differential transformer (LVDT). The pore water pressure was measured using a pore pressure transducer connected to the base of the samples. The volume change (i.e., amount of water leaving or entering a sample) during consolidation and shearing was recorded using a volume change device. The testing procedure consisted of three main stages, namely backpressure saturation, consolidation, and shearing. The shear strain rate applied was 0.30 mm/min. Backpressure saturation was performed in steps until the Skempton B value $\Delta {\text{u/}}\sigma_{3}^{\prime }$) of the samples exceeded 0.95. Figure 1 presents a schematic layout of the testing system.

The microfabric of the sand–clay mixtures containing different clay contents (i.e., 10, 20, and 30%) was examined by scanning electron microscopy (SEM). All samples were dried and coated with platinum to form electrical conducive specimens. The saturated samples were dried using the freeze–drying technique. Subsequently, the microfabric of the sand–clay mixtures was examined by SEM using JEOL JSM-7600F operated at 5–10 kV with a low magnification (100×) and a high resolution (3.0 nm). At the low magnification level, seeing more pores and repeated features is easier unlike at extra high magnifications.

In this study, the SEM-based classification system proposed by Collins and McGown^22^ and modified later by Collins^23^ was used to describe the microfabric of the sand–clay samples. This system included three main microfabric forms. These are elementary particles, particle assemblage, and pore space forms. Subforms and subdivisions were used to differentiate between different arrangements and define the forms in further detail. Particle assemblage is formed due to interactions between groups of clay flakes or clothed sand or silt grains. Pore spaces refer to voids within elementary arrangements or particle assemblage. Terms like edge to edge, edge to face, flocculated and dispersed particles are frequently used in this system. For granular soils the state of packing influences the fabric nature. Honeycombed or loose fabric can be formed due to low compaction effort. In their classification system, variable particle arrangements may take a parallel or random orientation. They introduced a form called “connectors” which refers to bridging or clothed grain-to-grain connections. All three SEM micrographs pointed to the presence of connectors with variable thickness and intensities (Fig. 8). Similarly, clay-coated particles can explain the minor changes in the frictional and shear resistance for a range of 10–30% clay content.

## Results and discussions

### Stress–strain response

**Figure 2:**
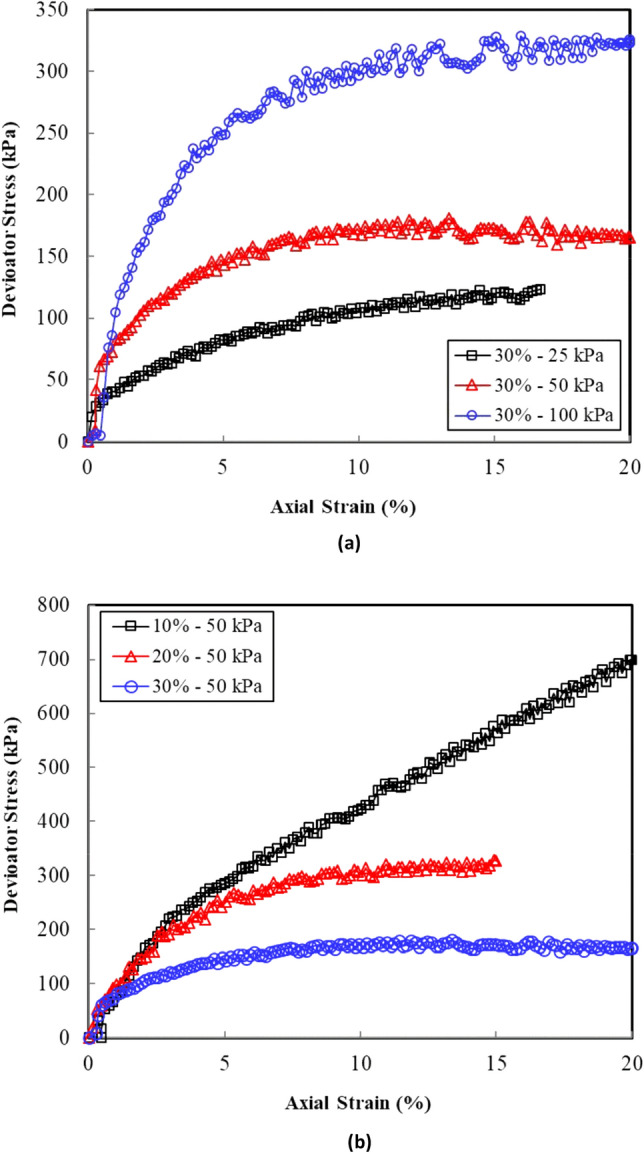
(**a**) Axial strain versus deviator stress for a selected sand–clay mixture. (**b**) Axial strain versus deviator stress for the sand–clay mixtures at 50 kPa cell pressure.

**Figure 3:**
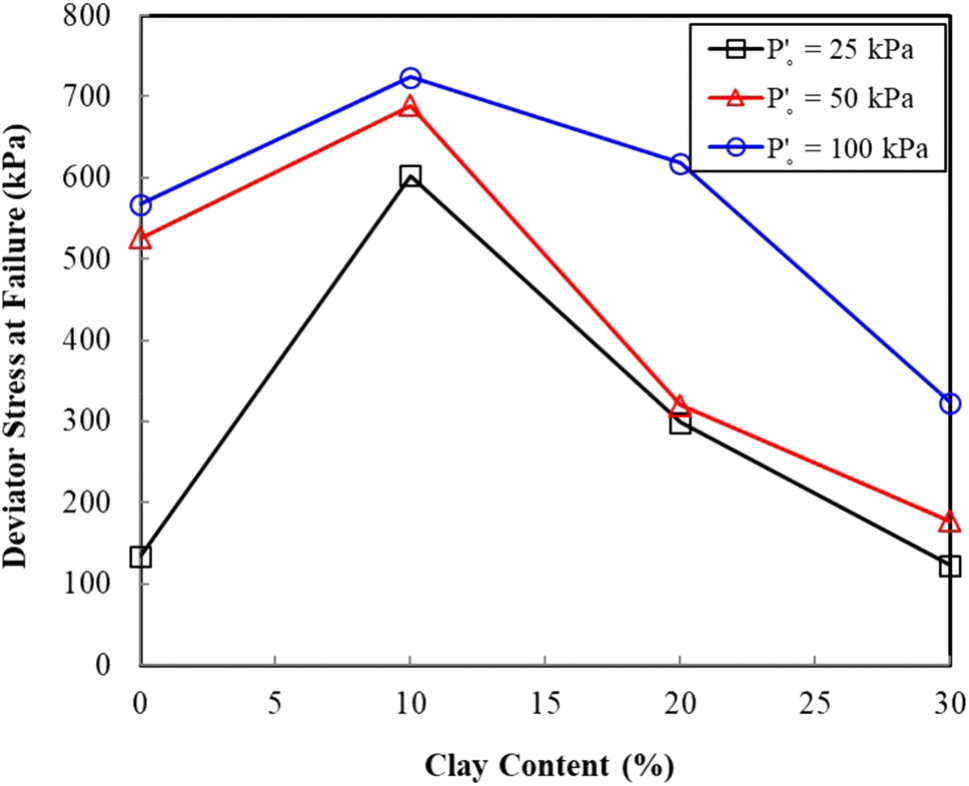
Variation of the deviator stress with clay content at failure with clay content.

Figure 2 shows a typical stress strain curve of the sand–clay mixture in terms of the deviator stress versus the axial strain relationship. These curves showed a hyperbolic response. Table 3 provides a summary of the deviator stress at failure hereinafter denoted as ($\sigma$_1_–$\sigma$_3_)_f_ for the different clay contents and confining pressures (Table 3). The deviator stress increased with the increase in the confining pressure, regardless of the clay content. Figure 3 illustrates a plot of the ($\sigma$_1_–$\sigma$_3_)_f_ variation with the clay content to visualize the effect of the clay content on the shear strength of the sand–clay mixtures. The deviator stress at failure increased in accordance with the increase in the clay content of up to 10%. A reverse trend was observed at failure for the clay contents greater than 10%, with the deviator stress decreasing in accordance with an increase in the clay content.

**Table 3:** Summary of the undrained shear strength tests.

Clay content	Initial effective confining pressure *p′*_*o*_ (kPa)	Deviator stress vs axial strain plots		Stress ratio vs axial strain plots	
		Deviator stress at failure ($\sigma$_1_–$\sigma$_3_)_*f*_ (kPa)	Steady-state strength $\tau$_ss_ (kPa)	Principle stress ratio at failure ($\sigma$_1_–$\sigma$_3_)_*f*_/($\sigma$_3_–u*w*)_*f*_	Strain at failure $\varepsilon$_f_ (%)
0%	25	134.4	132	10.09	1.03
	50	525.9	518.4	2.93	1.91
	100	567.5	567.5	2.21	2.80
10%	25	603.0	600	4.31	0.45
	50	689.2	689.2	2.93	1.71
	100	724.4	732.9	2.39	1.33
20%	25	298.9	294.4	3.69	1.04
	50	319.9	319.2	3.40	0.96
	100	618.1	578.0	2.92	1.91
30	25	122.5	119.7	2.98	1.04
	50	177.0	167.5	1.78	1.93
	100	323.0	323.0	2.38	2.24

**Figure 4:**
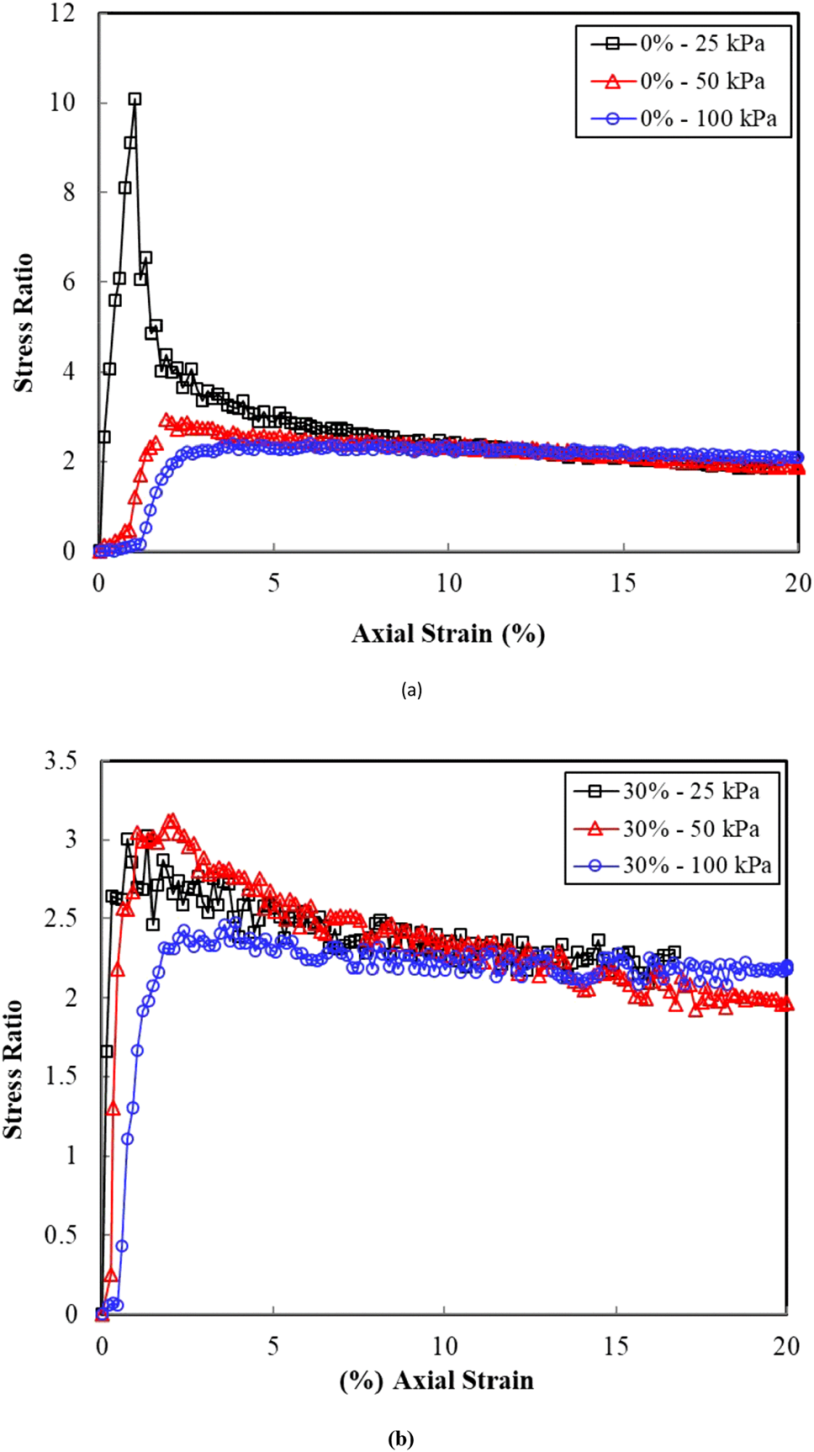
Stress–ratio versus axial strain for the sand clay mixtures: (**a**) clean sand and (**b**) 30% clay content.

Figure 4 shows an alternative representation of the stress strain behaviour of the sand–clay mixtures in terms of the effective stress ratio [($\sigma$_1_–$\sigma$_3_)/($\sigma$_3_–u_w_)] versus the axial strain, where $\sigma$_1_ is the axial vertical stress; $\sigma$_3_ is the radial cell pressure stress; and u_w_ is the pore water pressure. The effective stress ratio provides a better estimate of the shear strength behaviour of the sand–clay mixture compared to the deviator stress because the effective stress ratio at failure is a function of the pore water pressure measured during the undrained testing, whereas the deviator stress is not. Table 3 presents a summary of the stress ratio and the axial strain at failure for the tested sand–clay mixtures. The stress ratio at failure decreased in accordance with an increase in the clay content. Furthermore, the axial strain at failure ranged between 1% and 2.80%, and all the samples under a large strain (i.e., 20% strain) approached a stress ratio of 2.

### Pore pressure–deformation relationships

**Figure 5:**
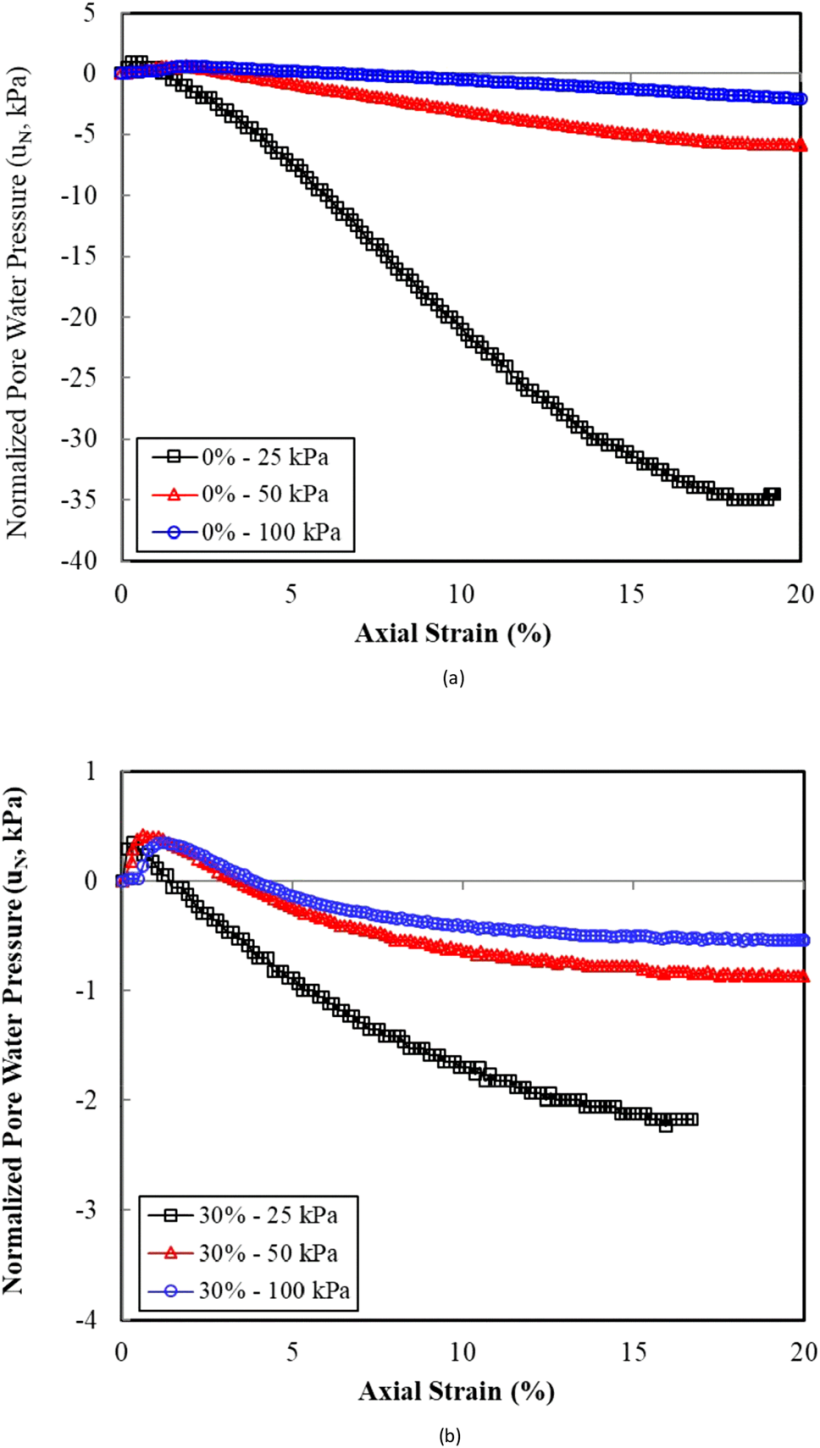
Effect of the clay content and the confining pressure on the normalized pore water pressure versus the axial strain response curves: (**a**) clean sand and (**b**) 30% clay content.

Figure 5 shows the variation in the normalized excess pore water pressure (u_N_), with the axial strain as a function of the clay content. The normalized excess pore water pressure is defined herein as the excess pore water pressure generated during the test divided by the initial effective confining pressure ($p_{o}^{\prime }$). Figure 5 depicts that all specimens initially underwent an increase in the pore water pressure (positive) under small axial strain values, followed by a significant decrease in the pore water pressure (negative). The decrease in the pore water pressures was indicative of the tendency of the samples with a dilatant behaviour. This dilative behaviour varied in accordance with the clay content (Fig. 5). The dilative tendency of the sand–clay specimens decreased as the clay content increased because of the relative contribution of the sand and clay components to the shear behaviour of the mixture under strain-controlled testing. In the samples with low clay contents, the sand grains dominated the shear behaviour causing the samples to show a higher tendency toward dilatation. The clayey matrix occupied the sand voids and contacts between the sand grains as the clay content increased, thereby increasing the compressibility of sample c and reducing the tendency to dilate. The compressibility is closely related to the hydraulic conductivity of the sand–clay mixtures^24^.

### Steady-state shear strength

The steady-state shear strength ($\tau$_ss_) represents the state at which a sample undergoes a change in the axial strain with no corresponding change in the shear strength. This typically occurs under large strain levels. In this state, a typical deviator stress–strain curve reaches a plateau of constant shear strength with the axial strain (Fig. 2). The steady-state shear strength is defined herein as the deviator stress at a large strain of 20%. In some tests, the stress–strain exhibited plateaus, especially in the sand–clay mixtures with clay contents of 0% and 10%. Table 3 summarizes the values of $\tau$_ss_ and the corresponding void ratio at the end of the consolidation stage (e_c_) prior to shearing.

**Figure 6:**
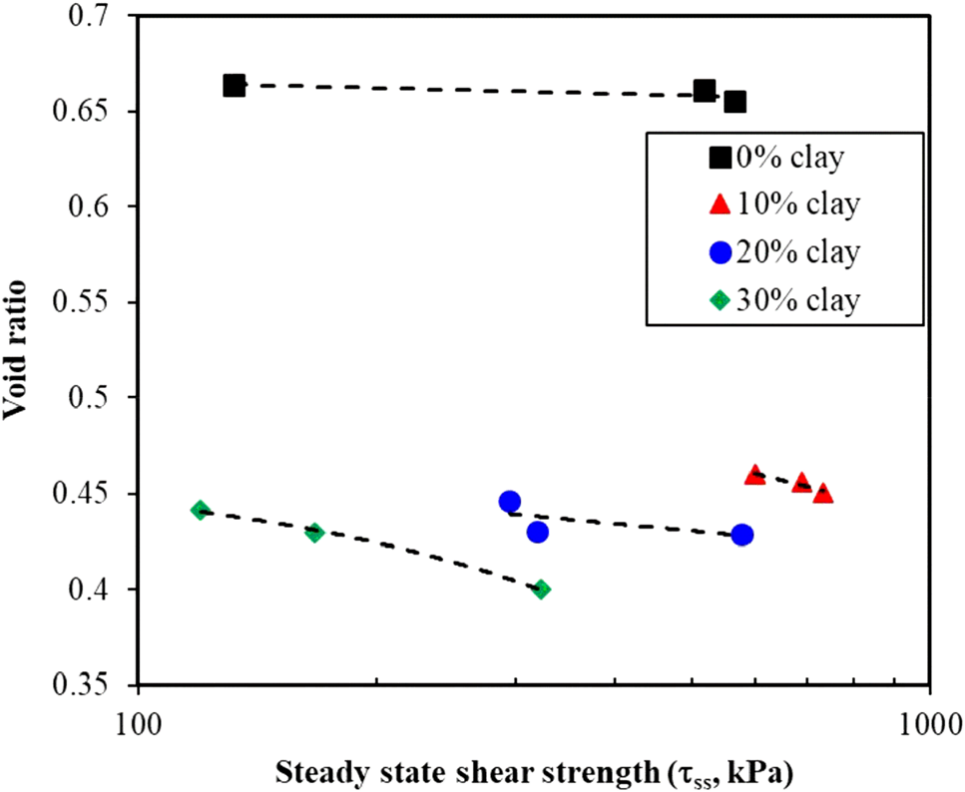
Steady-state shear strength versus void ratio for the sand–clay mixtures with different clay contents.

Given the hyperbolic trend of the stress strain response curves (Fig. 2), the steady-state stress was almost the same as the deviator stress at failure. Figure 6 presents the steady-state stress variation with e_c_ for the sand–clay mixtures with different clay contents. The clay contents considered showed a significant shift in the position of the steady-state line with respect to e_c_. However, a less marked change in the steady-state shear strength was observed. The variation in the void ratio versus the steady-state shear strength indicated a mild slope for the pure sand, 10% clay, and 20% clay. Moreover, the slope was found steeper for the 30% clay content.

### Effective stress path

**Figure 7:**
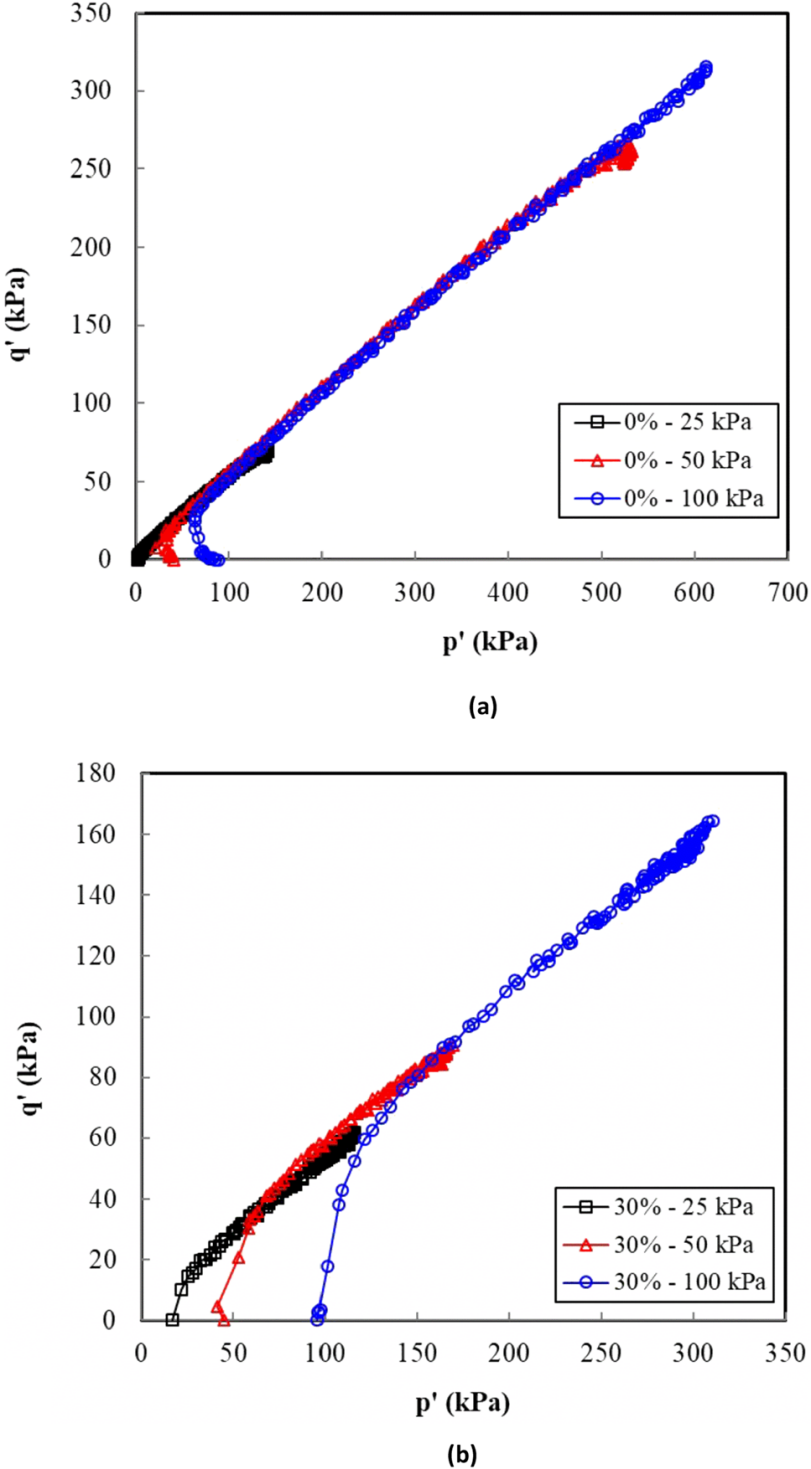
Effective stress path (*p′*–*q*) for the sand–clay mixtures: (**a**) clean sand and (**b**) 30% clay content.

The change in the shear strength behaviour of the compacted sand–clay mixtures is represented herein by an effective stress path. Figure 7 illustrates the effective stress paths of the tested samples. The mean stress *p′* and *q′* in the aforementioned figures are defined as given in Eqs. (1) and (2).

1 $$p^{\prime } = \frac{{(\sigma_{1}^{\prime } + \sigma_{3}^{\prime } )}}{2}$$

2 $$q^{\prime } = \frac{{(\sigma_{1}^{\prime } - \sigma_{3}^{\prime } )}}{2}$$

**Figure 8:**
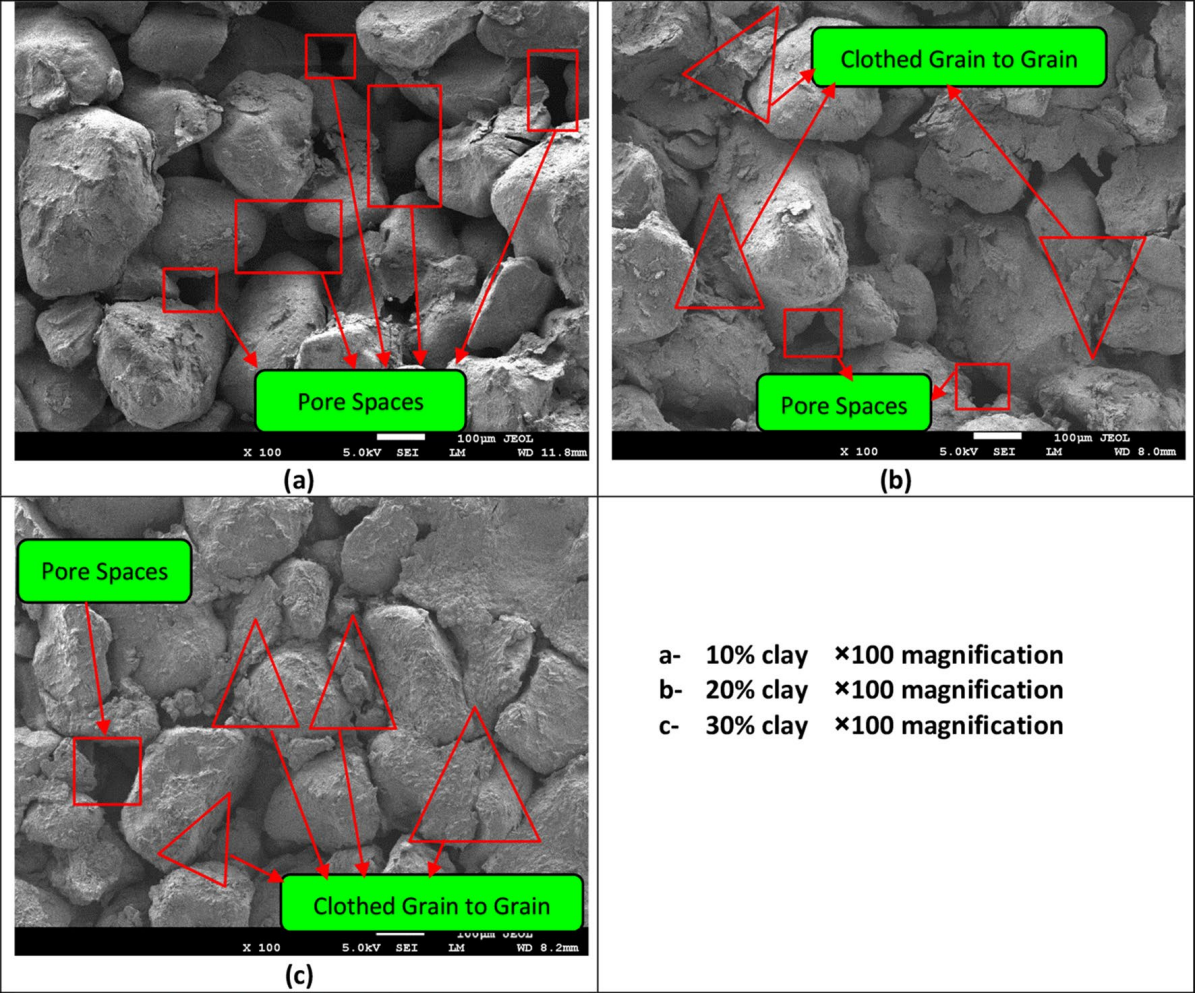
Sand–clay fabric as shown in the scanning electron microscope (SEM).

For clean sand (Fig. 7), the samples underwent a contractive behaviour followed by a dilative behaviour at all confining pressures. The contractive behaviour was marked by a reduction in *p′* in accordance with an increase in *q′*. This contractive behaviour became less marked as the clay content increased (Fig. 7), especially when high confining pressures were applied (i.e., 100 kPa). Figure 7 depicts that the deviator stress and the effective mean stress at failure in each test series failed along a line defined as the failure line. The plots of the stress ratio versus the axial strain (Fig. 4) showed a steady-state deformation (i.e., a change in the axial strain without a change in the deviator stress); hence, the failure line can be considered as a critical-state line (CSL). The critical-state friction angle ($\phi_{{{\text{cr}}}}$) was evaluated as shown in Eq. 3^21^. Fig. 8 presents the sand - clay fabric as observed in the scanning electron microscope (SEM).

3 $$M = \frac{{6\sin \phi^{\prime } }}{{3 - \sin \phi^{\prime } }}$$

where, M is the CSL slope. Table 4 apparently shows that the clay content had a negligible effect on $\phi_{{{\text{cr}}}}$_._

**Table 4:** Effect of the clay content on the slope of the failure line and the critical-state friction angle.

Clay content (%)	Slope of failure line (M)	Critical state friction angle φ^′^, degrees
0%	0.493	13.17
10%	0.501	13.37
20%	0.479	12.81
30%	0.476	12.74

## Conclusion

This study experimentally investigated the undrained shear strength characteristics of saturated sand–clay mixtures. The specific conclusions deduced from this study are as follows:

- The undrained behaviour of the sand–clay mixtures is best interpreted using the stress ratio versus axial strain representation because the effective stress ratio at failure is a function of the pore water pressure measured during the undrained testing, whereas the deviator stress is not.
- The pore water pressure generation curves (i.e., normalized pore water pressure versus axial strain) indicated the tendency of samples to contract during the early loading stages followed by the tendency to dilate, which is manifested in the form of a negative pore water pressure. The magnitude of the tendency to dilate decreases with the increase in the clay content and effective confining pressure.
- The test results revealed that the clay content significantly affect the steady-state line location.
- The effective stress paths of the saturated sand–clay mixtures revealed the initial tendency of a contractive behaviour for the sand–clay mixtures with a clay content of less than 10%. Furthermore, the clay content had a negligible effect on the slope of the failure line (M) and the critical-state friction angle. Particle coating connectors can reduce the friction and interlocking nature of the coarse-grained content of the mixture.

## Data Availability

The data used to support the findings of this study are available from the corresponding author upon request.
